# Classifying Alzheimer's Disease Using Audio and Text-Based Representations of Speech

**DOI:** 10.3389/fpsyg.2020.624137

**Published:** 2021-01-15

**Authors:** R'mani Haulcy, James Glass

**Affiliations:** Computer Science and Artificial Intelligence Laboratory, Massachusetts Institute of Technology, Cambridge, MA, United States

**Keywords:** Alzheimer's disease, dementia detection, speech, BERT, i-vectors, x-vectors, word vectors, MMSE prediction

## Abstract

Alzheimer's Disease (AD) is a form of dementia that affects the memory, cognition, and motor skills of patients. Extensive research has been done to develop accessible, cost-effective, and non-invasive techniques for the automatic detection of AD. Previous research has shown that speech can be used to distinguish between healthy patients and afflicted patients. In this paper, the ADReSS dataset, a dataset balanced by gender and age, was used to automatically classify AD from spontaneous speech. The performance of five classifiers, as well as a convolutional neural network and long short-term memory network, was compared when trained on audio features (i-vectors and x-vectors) and text features (word vectors, BERT embeddings, LIWC features, and CLAN features). The same audio and text features were used to train five regression models to predict the Mini-Mental State Examination score for each patient, a score that has a maximum value of 30. The top-performing classification models were the support vector machine and random forest classifiers trained on BERT embeddings, which both achieved an accuracy of 85.4% on the test set. The best-performing regression model was the gradient boosting regression model trained on BERT embeddings and CLAN features, which had a root mean squared error of 4.56 on the test set. The performance on both tasks illustrates the feasibility of using speech to classify AD and predict neuropsychological scores.

## 1. Introduction

Alzheimer's Disease (AD) is a progressive, neurodegenerative disease that affects the lives of more than 5 million Americans every year. The number of Americans living with AD is expected to be more than double that number by 2050. AD is a deadly and costly disease that has negative emotional, mental, and physical implications for those afflicted with the disease and their loved ones (Alzheimer's Association, 2019).

There is currently no cure for AD (Yadav, 2019) and early detection is imperative for effective intervention to occur (De Roeck et al., 2019). Currently, AD is diagnosed using PET imaging and cerebrospinal fluid exams to measure the concentration of amyloid plaques in the brain, a costly and invasive process (Land and Schaffer, 2020). A more cost-effective, non-invasive and easily-accessible technique is needed for detecting AD.

Previous research has shown that speech can be used to distinguish between healthy and AD patients (Pulido et al., 2020). Some researchers have focused on developing new machine learning model architectures to improve detection (Chen et al., 2019; Chien et al., 2019; Liu et al., 2020), while others have used language models (Guo et al., 2019) to classify AD. Others have focused on trying to extract acoustic and text features that capture information indicative of AD. These features include non-verbal features, such as the length of segments and the amount of silence (König et al., 2015). Other researchers have used linguistic and audio features extracted from English speech (Fraser et al., 2016; Gosztolya et al., 2019), as well as Turkish speech (Khodabakhsh et al., 2015). Prosodic features have been extracted from English speech (Ossewaarde et al., 2019; Nagumo et al., 2020; Qiao et al., 2020) and German speech (Weiner et al., 2016) to classify AD, and so have paralinguistic acoustic features (Haider et al., 2019). Other researchers have chosen to focus on the type of speech data that is used instead of the type of model or type of features and have used speech from people performing multiple tasks to improve generalizability (Balagopalan et al., 2018). This provides a brief summary of the work that has been done in the past few years. A more extensive review of the background literature can be found in the review paper of de la Fuente Garcia et al. (2020).

Although promising research has been done, the datasets that have been used are often imbalanced and vary across studies, making it difficult to compare the effectiveness of different modalities. Two recent review papers (Voleti et al., 2019; de la Fuente Garcia et al., 2020) explain that an important future direction for the detection of cognitive impairment is providing a balanced, standardized dataset that will allow researchers to compare the effectiveness of different classification techniques and feature extraction methods. This is what the ADReSS challenge attempted to do. The ADReSS challenge provided an opportunity for different techniques to be performed on a balanced dataset that alleviated the common biases associated with other AD datasets and allowed those techniques to be directly compared.

Previous work has been done using the ADReSS dataset. Some researchers only participated in the AD classification task (Edwards et al., 2020; Pompili et al., 2020; Yuan et al., 2020), others only participated in the Mini-Mental State Examination (MMSE) prediction task (Farzana and Parde, 2020), and others participated in both tasks (Balagopalan et al., 2020; Cummins et al., 2020; Koo et al., 2020; Luz et al., 2020; Martinc and Pollak, 2020; Pappagari et al., 2020; Rohanian et al., 2020; Sarawgi et al., 2020; Searle et al., 2020; Syed et al., 2020). The best performance on the AD classification task was achieved by Yuan et al. (2020), who obtained an accuracy of 89.6% on the test set using linguistic features extracted from the transcripts, as well as encoded pauses. The best performance on the MMSE prediction task was achieved by Koo et al. (2020), who obtained a root mean squared error (RMSE) of 3.747 using a combination of acoustic and textual features.

In this paper, audio features (i-vectors and x-vectors) and text features (word vectors, BERT embeddings, LIWC features, and CLAN features) were extracted from the data and used to train several classifiers, neural networks, and regression models to detect AD and predict MMSE scores. I-vectors and x-vectors, originally intended to be used for speaker verification, have been shown to be effective for detecting AD (López et al., 2019) and other neurodegenerative diseases, such as Parkinson's Disease (Botelho et al., 2020; Moro-Velazquez et al., 2020). Word vectors have also been shown to be useful for detecting AD (Hong et al., 2019). I-vectors, x-vectors, and BERT embeddings have been used with the ADReSS dataset to classify AD (Pompili et al., 2020; Yuan et al., 2020) and predict MMSE scores (Balagopalan et al., 2020). Pompili et al. (2020) used the same audio features that we used and also used BERT embeddings, but they did not apply their techniques to the MMSE prediction task and their best fusion model obtained lower performance on the classification task than our best model. The difference between our work and the work of Balagopalan et al. (2020) and Yuan et al. (2020) is that they finetuned a pre-trained BERT model on the ADReSS data and used that model for classification and regression, whereas we used a pre-trained BERT model as a feature extractor and then trained different classifiers and regressors on the extracted BERT embeddings.

CLAN features were used in the baseline paper (Luz et al., 2020) and were combined with BERT embeddings in this paper to explore whether performance improved. Lastly, LIWC features have been used to distinguish between AD patients and healthy controls in the past (Shibata et al., 2016) but the dataset was very small (nine AD patients and nine healthy controls), and to our knowledge, literature using LIWC for Alzheimer's detection is limited. However, LIWC features have been used to analyze other aspects of mental health (Tausczik and Pennebaker, 2010) and may be useful in the field of AD. For these reasons, we wanted to further explore whether LIWC features could be useful for AD detection and MMSE prediction. Even though our results do not out-perform the best performance on the classification and MMSE prediction tasks, the approaches we employ are different than previous approaches, which provides additional insight into which techniques are best for AD classification and MMSE prediction.

## 2. Materials and Methods

### 2.1. ADReSS Dataset

The ADReSS challenge dataset consists of audio recordings, transcripts, and metadata (age, gender, and MMSE score) for non-AD and AD patients. The dataset is balanced by age, gender, and number of non-AD vs. AD patients, with there being 78 patients for each class. The audio recordings are of each patient completing the cookie theft picture description task, where each participant describes what they see in the cookie theft image. This task has been used for decades to diagnose and compare AD and non-AD patients (Cooper, 1990; Mendez and Ashla-Mendez, 1991; Giles et al., 1996; Bschor et al., 2001; Mackenzie et al., 2007; Choi, 2009; Hernández-Doḿınguez et al., 2018; Mueller et al., 2018), as well as patients with other forms of cognitive impairment, and was originally designed as part of an aphasia examination (Goodglass and Kaplan, 1983).

Normalized audio chunks were provided for each speaker, in which a voice activity detection (VAD) system was applied to each patient's recording to split it into several chunks. The VAD system used a log energy threshold value to detect the sections of the audio that contained speech by ignoring sounds below a certain threshold. A 65 dB log energy threshold value was used, along with a maximum duration of 10 s per chunk. Volume normalization involves changing the overall volume of an audio file to reach a certain volume level. There was some variation in the recording environment for each audio file, such as microphone placement, which lead to variation in the volume levels for different recordings. The volume of each chunk was normalized relative to its largest value to remove as much variation from the recordings as possible. Each patient had an average of 25 normalized audio chunks, with a standard deviation of 13 chunks. The CHAT coding system (MacWhinney, 2014) was used to create the transcripts.

The ADReSS dataset is a subset of the Pitt corpus (Becker et al., 1994), which is a dataset that contains 208 patients with possible and probable AD, 104 healthy patients, and 85 patients with an unknown diagnosis. The dataset consists of transcripts and recorded responses from the participants for the cookie theft picture description task, a word fluency task, and a story recall task. In order to provide additional in-domain data for training some of the feature extractors, the cookie theft data for patients not included in the ADReSS dataset was separated from the Pitt corpus and used for pre-training. Normalized audio chunks for this data were created using the steps mentioned above. The pre-training process is described in greater detail in section 2.2.2.

The age and gender distributions, along with the average MMSE scores, average years of education, and corresponding standard deviations, for the training and test sets, can be seen in Tables 1, 2. Education information was not provided with the ADReSS dataset. However, the Pitt corpus did have education information and was cross-referenced with the ADReSS dataset to determine which patients overlapped and to extract each patient's education information. A total of 108 patients (54 non-AD and 54 AD) were selected from the full dataset to create the training set, and the remaining 48 patients (24 non-AD and 24 AD) were used for the test set. For both the training and test sets, an equal number of AD and non-AD patients were included for each age group and the number of male and female AD and non-AD patients was the same for each age group. For the training set, the average MMSE score for the AD patients was 17.0 and the average MMSE score for the non-AD patients was 29.1. The average years of education were 11.9 and 14.3 for the AD and non-AD patients, respectively. For the test set, the AD patients had an average MMSE score of 19.5 and the non-AD patients had an average MMSE score of 28.8. The average years of education were 12.8 and 13.2 for the AD and non-AD patients, respectively.

**Table 1:** Age and gender details for patients in the training set, as well as the average MMSE scores, average years of education, and corresponding standard deviations (sd), for the patients in each age interval.

	**AD**				**Non-AD**			
**Age interval**	**Male**	**Female**	**MMSE (sd)**	**Educ. (sd)**	**Male**	**Female**	**MMSE (sd)**	**Educ. (sd)**
[50, 55)	1	0	30.0 (n/a)	12.0 (n/a)	1	0	29.0 (n/a)	12.0 (n/a)
[55, 60)	5	4	16.3 (4.9)	12.4 (1.7)	5	4	29.0 (1.3)	15.8 (2.8)
[60, 65)	3	6	18.3 (6.1)	12.5 (2.1)	3	6	29.3 (1.3)	13.1 (2.3)
[65, 70)	6	10	16.9 (5.8)	12.8 (2.0)	6	10	29.1 (0.9)	13.8 (3.1)
[70, 75)	6	8	15.8 (4.5)	10.4 (2.6)	6	8	29.1 (0.8)	14.9 (3.4)
[75, 80)	3	2	17.2 (5.4)	10.6 (2.7)	3	2	28.8 (0.4)	14.2 (3.7)
Full set	24	30	17.0 (5.5)	11.9 (2.4)	24	30	29.1 (1.0)	14.3 (3.1)

**Table 2:** Age and gender details for patients in the test set, as well as the average MMSE scores, average years of education, and corresponding standard deviations (sd), for the patients in each age interval.

	**AD**				**Non-AD**			
**Age interval**	**Male**	**Female**	**MMSE (sd)**	**Educ. (sd)**	**Male**	**Female**	**MMSE (sd)**	**Educ. (sd)**
[50, 55)	1	0	23.0 (n/a)	20.0 (n/a)	1	0	28.0 (n/a)	12.0 (n/a)
[55, 60)	2	2	18.7 (1.0)	12.5 (1.0)	2	2	28.5 (1.2)	13.7 (2.1)
[60, 65)	1	3	14.7 (3.7)	13.2 (2.2)	1	3	28.7 (0.9)	12.2 (0.5)
[65, 70)	3	4	23.2 (4.0)	11.7 (1.9)	3	4	29.4 (0.7)	13.3 (1.4)
[70, 75)	3	3	17.3 (6.9)	12.8 (3.6)	3	3	28.0 (2.4)	13.2 (1.8)
[75, 80)	1	1	21.5 (6.3)	13.0 (1.4)	1	1	30.0 (0.0)	14.0 (2.8)
Full set	11	13	19.5 (5.3)	12.8 (2.7)	11	13	28.8 (1.5)	13.2 (1.6)

### 2.2. Feature Extraction

#### 2.2.1. Text Features: fastText Word Vectors, BERT Embeddings, LIWC, and CLAN Features

FastText is an open-source library that is used to classify text and learn text representations. A fastText model pre-trained on Common Crawl and Wikipedia was used to extract word vectors (Grave et al., 2018) from the transcripts of each speaker. PyLangAcq (Lee et al., 2016), a Python library designed to handle CHAT transcripts, was used to extract the sentences from the CHAT transcript of each participant. A 100-dimensional word vector was computed for each word in each sentence, including punctuation. A dimension of 100 was chosen because this was the value recommended on the fastText website and 100 was compatible with the size of the pre-trained model. The longest sentence had a total of 47 words. For this reason, every sentence was padded to a length of 47, resulting in a (47, 100) representation for each utterance.

Bidirectional Encoder Representations from Transformers (BERT) (Devlin et al., 2018) models are text classification models that have achieved state-of-the-art results on a wide variety of natural language processing tasks and they provide high-level language representations called embeddings. Embeddings are vector representations of words or phrases and are useful for representing language because the embeddings often capture information that is universal across different tasks. Keras BERT was used to load an official, pre-trained BERT model and that model was used to extract embeddings of shape (*x*,768) for each utterance in the transcript of each speaker, where *x* depends on the length of the input. After embeddings were extracted for each utterance, the largest embedding had an *x* value of 60. For this reason, the remaining embeddings were padded to be the same shape, resulting in a (60,768) embedding for each utterance. For both the word vectors and the BERT embeddings, features were extracted at the utterance level, resulting in a total of 1,492 embeddings in the training set and 590 embeddings in the test set.

Linguistic Inquiry and Word Count (LIWC) (Tausczik and Pennebaker, 2010) features were also extracted from the transcripts of each speaker. The LIWC program takes in a transcript and outputs a 93-dimensional vector consisting of word counts for different emotional and psychological categories, such as emotional tone, authenticity, and clout, to name a few. The Computerized Language Analysis (CLAN) program was also used to extract linguistic features from the transcripts of each speaker. The EVAL function was used to extract summary data, including duration, percentage of word errors, number of repetitions, etc. This extraction resulted in a 34-dimensional vector for each speaker. The CLAN features were used as linguistic features in the baseline paper (Luz et al., 2020). In this paper, the CLAN features were combined with the BERT embeddings to explore whether combining the features improved performance. Both the LIWC and CLAN features were extracted at the subject-level, resulting in 108 vectors in the training set and 54 vectors in the test set.

#### 2.2.2. Audio Features: I-Vectors and X-Vectors

VoxCeleb 1 and 2 (Nagrani et al., 2017) are datasets consisting of speech that was extracted from YouTube videos of interviews with celebrities. I-vector and x-vector systems (Snyder et al., 2017, 2018) pre-trained on VoxCeleb 1 and 2 were used to extract i-vectors and x-vectors from the challenge data. The i-vector and x-vector systems were built using Kaldi (Povey et al., 2011), which is a toolkit that is used for speech recognition. The pre-trained VoxCeleb models were also used to train additional extractors using the original Kaldi recipes. The original VoxCeleb models were used to initialize the i-vector and x-vector extractors and then those extractors were trained on the remaining in-domain Pitt data. I-vector and x-vector extractors were also trained on only the in-domain Pitt data to explore whether a small amount of in-domain data is better for performance than a large amount of out-of-domain data. For each type of extractor, the normalized audio chunks provided with the challenge dataset were first resampled with a sampling rate of 16kHz, a single channel, and 16 bits, to match the configuration of the VoxCeleb data. The Kaldi toolkit was then used to extract the Mel-frequency cepstral coefficients (MFCCs), compute the voice activation detection (VAD) decision, and extract the i-vectors and x-vectors. The x-vectors had a length of 512, while the i-vectors had a length of 400. There were a total of 2,834 i-vectors and 2,834 x-vectors, one i-vector and x-vector for each normalized audio chunk.

## 2.3. Experimental Approach

### 2.3.1. Classifiers

Five classifiers were trained on the text and audio features explained in sections 2.2.1 and 2.2.2: linear discriminant analysis (LDA), the decision tree (DT) classifier, the k-nearest neighbors classifier with the number of neighbors set to 1 (1NN), a support vector machine (SVM) with a linear kernel and regularization parameter set to 0.1, and a random forest (RF) classifier. The classifiers were implemented in Python using the scikit-learn library. The word vectors and BERT embeddings were averaged before being used to train the scikit-learn classifiers, resulting in utterances represented by 100-dimensional vectors and 768-dimensional vectors, respectively. When the LIWC and CLAN features were combined with the averaged BERT embeddings, the subject-level LIWC/CLAN vector was concatenated with each utterance-level BERT embedding belonging to that subject. Standard scaling is commonly applied to data before using machine learning estimators to avoid the poor performance that is sometimes seen when the features are not normally distributed (i.e., Gaussian with a mean of 0 and unit variance). Because we were combining different types of features with different data distributions, standard scaling was applied to the features after the LIWC/CLAN vectors were concatenated with the BERT embeddings so that the data would be normally distributed before training and testing.

### 2.3.2. Regressors

Five regression models were also trained on the text and audio features explained in sections 2.2.1 and 2.2.2 for the MMSE prediction task: linear regression (LR), decision tree (DT) regressor, k-nearest neighbor regressor with the number of neighbors set to 1 (1NN), support vector machine (SVM), and a gradient-boosting regressor (grad-boost). The regression models were implemented in Python using the scikit-learn library. Just as with the classifiers, the word vectors and BERT embeddings were averaged before being used to train the scikit-learn regressors. When the LIWC and CLAN features were combined with the BERT embeddings, the subject-level LIWC/CLAN vector was concatenated with each utterance-level BERT embedding belonging to that subject, and after the features were concatenated, standard scaling was applied.

### 2.3.3. Dimensionality Reduction

The classifiers and regressors mentioned in sections 2.3.1 and 2.3.2 were trained with different dimensionality reduction techniques to see if applying dimensionality reduction improves performance. Feature sets were created with no dimensionality reduction, with LDA, and with principal component analysis (PCA), and each classifier was trained on each feature set to see what effect dimensionality reduction had on performance. The dimensionality reduction techniques were applied to all of the audio and text features. When LDA was applied, the features were reduced to 1 dimension for the classification task and 23 dimensions for the regression task. With PCA, different dimension values were selected manually. The best results and corresponding dimension values can be seen in the Results section.

### 2.3.4. Neural Networks

**Figure 1:**
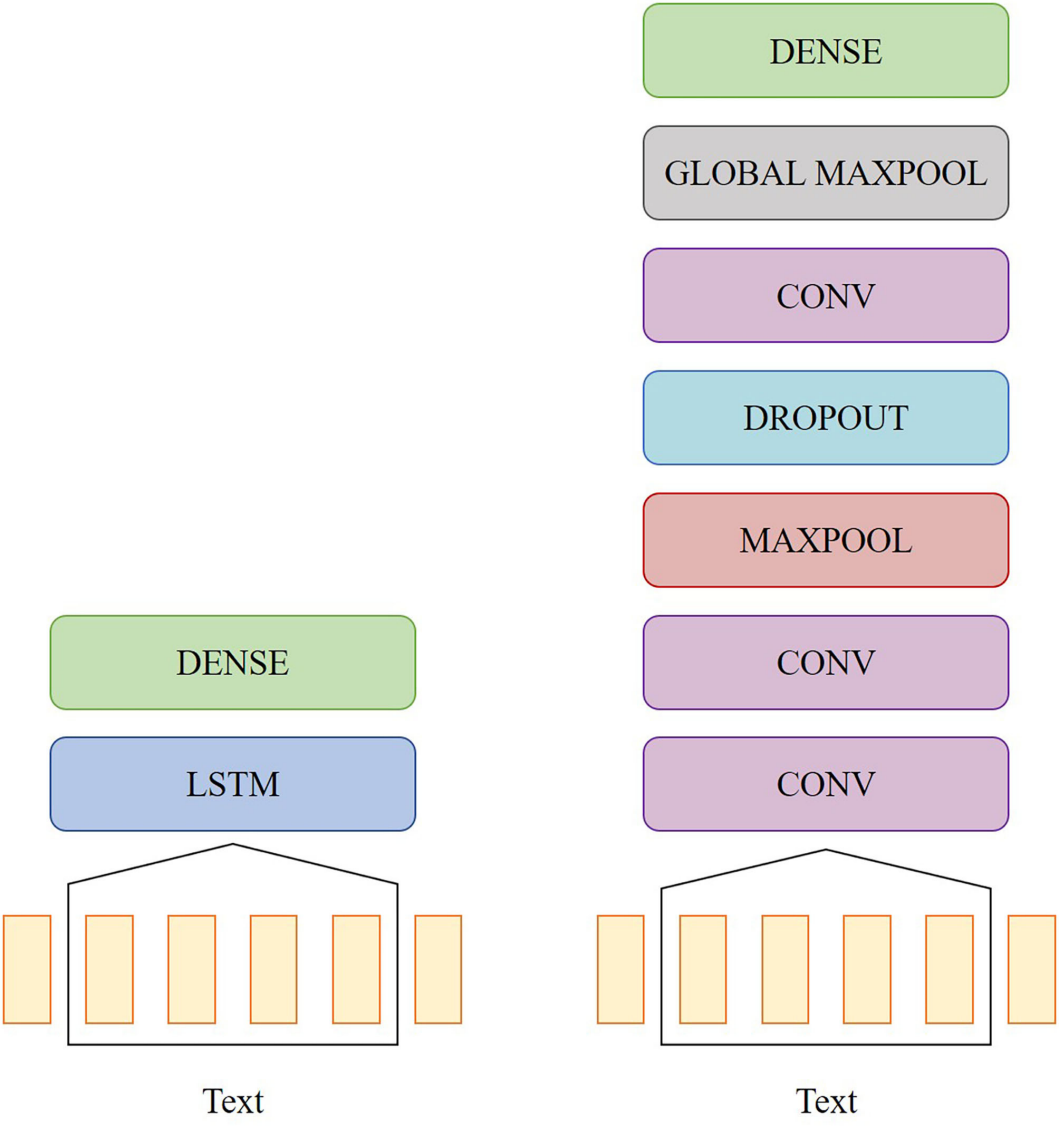
Diagrams of the network topology for the LSTM model (left) and the CNN model (right).

A bidirectional long short-term memory (LSTM) network and a convolutional neural network (CNN) were also trained on the word vectors to see if the neural networks could extract some temporal information that would lead to better performance compared to the classifiers mentioned in section 2.3.1. The topologies of the two networks are shown in Figure 1. The LSTM model had one bidirectional LSTM layer with eight units, a dropout rate of 0.2, and a recurrent dropout rate of 0.2. The CNN model had the following layers: three 2D convolution layers with 32, 64, and 128 filters, respectively, rectified linear unit (ReLu) activation and a kernel size of 3, one 2D max pooling layer with a pool size of 3, one dropout layer with a rate of 0.5, and one 2D global max pooling layer. For both models, the output was passed into a dense layer with sigmoid activation. Both models were implemented in Python using Keras and were trained with an Adam optimizer. The CNN was trained with a learning rate of 0.001, and the LSTM was trained with a learning rate of 0.01.

## 3. Results

### 3.1. Classification

#### 3.1.1. Cross-Validation

In order to stay consistent with the baseline paper, each of the classifiers and neural networks were evaluated on the challenge training set using leave-one-subject-out (LOSO) cross-validation, where there was no speaker overlap between the training and test sets for each split. Each model was trained and tested at the utterance level, where each utterance was classified as belonging to a patient with or without AD. Then majority vote (MV) classification was used to assign a label to each speaker based on the label that was assigned most to the speaker's utterances.

The MV classification accuracy (the number of correctly classified speakers divided by the total number of speakers), for each feature type can be seen in Table 3. The accuracies are presented as decimals and are rounded to 3 decimal places to match the form of the accuracies in the baseline paper. For all of the features, the LDA classifier trained on LDA-reduced features performed the same as the LDA classifier trained on features with no dimensionality reduction. Although there was no difference in performance, results are included for completeness.

**Table 3:** LOSO accuracies for each of the classifiers. The best-performing models for each feature type are red.

**Features**	**Dim. Red. (n_comp)**	**LDA**	**DT**	**1NN**	**SVM**	**RF**
LIWC	None	0.741	0.593	0.620	0.833	0.778
	LDA (1)	0.741	0.750	0.750	0.731	0.750
	PCA (20)	0.778	0.620	0.704	0.787	0.759
BERT	None	0.713	0.676	0.787	0.796	0.769
	LDA (1)	0.713	0.657	0.667	0.713	0.657
	PCA (2)	0.630	0.648	0.602	0.546	0.694
	PCA (20)	0.750	0.713	0.722	0.769	0.796
BERT + LIWC	None	0.750	0.657	0.667	0.824	0.806
	LDA (1)	0.750	0.731	0.731	0.741	0.731
	PCA (20)	0.824	0.620	0.657	0.824	0.796
BERT + CLAN	None	0.778	0.657	0.759	0.824	0.750
	LDA (1)	0.778	0.769	0.769	0.787	0.769
	PCA (20)	0.824	0.630	0.657	0.898	0.778
BERT + LIWC + CLAN	None	0.593	0.731	0.713	0.815	0.806
	LDA (1)	0.593	0.611	0.611	0.593	0.611
	PCA (20)	0.833	0.731	0.713	0.815	0.787
word vectors	None	0.759	0.731	0.694	0.259	0.694
	LDA (1)	0.759	0.741	0.731	0.759	0.759
	PCA (2)	0.676	0.620	0.565	0.259	0.620
	PCA (70)	0.796	0.648	0.759	0.796	0.787
i-vectors (VoxCeleb)	None	0.574	0.423	0.454	0.574	0.500
	LDA (1)	0.574	0.500	0.500	0.574	0.500
	PCA (2)	0.491	0.500	0.602	0.519	0.491
	PCA (10)	0.528	0.556	0.546	0.491	0.528
i-vectors (Pitt)	None	0.528	0.491	0.500	0.509	0.593
	LDA (1)	0.528	0.537	0.537	0.537	0.537
	PCA (2)	0.463	0.500	0.528	0.343	0.546
	PCA (20)	0.565	0.537	0.528	0.565	0.565
i-vectors (VoxCeleb + Pitt)	None	0.528	0.509	0.500	0.528	0.556
	LDA (1)	0.528	0.519	0.519	0.528	0.519
	PCA (20)	0.519	0.528	0.574	0.472	0.620
x-vectors (VoxCeleb)	None	0.583	0.620	0.509	0.546	0.574
	LDA (1)	0.583	0.593	0.593	0.583	0.593
	PCA (2)	0.472	0.537	0.491	0.454	0.491
	PCA (40)	0.639	0.583	0.528	0.639	0.583
x-vectors (Pitt)	None	0.546	0.546	0.472	0.528	0.481
	LDA (1)	0.546	0.500	0.500	0.537	0.500
	PCA (40)	0.537	0.481	0.435	0.528	0.491
x-vectors (VoxCeleb + Pitt)	None	0.639	0.602	0.519	0.620	0.509
	LDA (1)	0.639	0.509	0.509	0.630	0.509
	PCA (40)	0.657	0.574	0.546	0.593	0.593

The LSTM model trained on word vectors had an average accuracy of **0.787**, while the CNN model had an average accuracy of **0.704**. The highest-performing classifier trained on text features was the SVM classifier trained on a combination of BERT embeddings and CLAN features with PCA dimensionality reduction applied, which had an average accuracy of 0.898. The highest-performing classifier trained on audio features was the LDA classifier trained on x-vectors that were extracted using a system that was pre-trained on VoxCeleb and in-domain Pitt data. PCA dimensionality reduction was applied and the classifier had an average accuracy of 0.657.

The highest-performing classifiers for each feature type, except for the classifiers trained on x-vectors that were extracted from a system trained on just Pitt data, performed better than the highest-performing audio and text baseline classifiers that were evaluated using LOSO on the training set, which had an average accuracy of 0.565 and 0.768, respectively (Luz et al., 2020).

#### 3.1.2. Held-Out Test Set

The MV classification accuracies on the test set for each of the classifiers can be seen in Table 4. The highest-performing text classifiers were the SVM classifier with no dimensionality reduction and the RF classifier with PCA dimensionality reduction, both trained on BERT embeddings. Both classifiers had an average accuracy of 0.854. The highest-performing audio classifier was the 1NN classifier trained on i-vectors that were extracted using systems pre-trained on VoxCeleb with PCA dimensionality reduction applied, which had an average accuracy of 0.563.

**Table 4:** Accuracies for classifiers evaluated on the test set. The test set results for the best-performing models during cross-validation are red.

**Features**	**Dim. Red. (n_comp)**	**LDA**	**DT**	**1NN**	**SVM**	**RF**
LIWC	None	0.583	0.708	0.583	0.688	0.812
	LDA (1)	0.583	0.583	0.583	0.583	0.583
	PCA (20)	0.771	0.646	0.583	0.792	0.667
BERT	None	0.604	0.708	0.771	0.854	0.750
	LDA (1)	0.604	0.604	0.646	0.604	0.604
	PCA (2)	0.688	0.562	0.542	0.729	0.625
	PCA (20)	0.833	0.646	0.750	0.812	0.854
BERT + LIWC	None	0.583	0.667	0.688	0.729	0.812
	LDA (1)	0.583	0.583	0.583	0.583	0.583
	PCA (20)	0.792	0.708	0.771	0.771	0.792
BERT + CLAN	None	0.729	0.750	0.771	0.812	0.812
	LDA (1)	0.729	0.708	0.708	0.708	0.708
	PCA (20)	0.729	0.708	0.667	0.771	0.792
BERT + LIWC + CLAN	None	0.625	0.688	0.750	0.750	0.812
	LDA (1)	0.625	0.667	0.667	0.625	0.667
	PCA (20)	0.812	0.604	0.729	0.812	0.812
word vectors	None	0.813	0.688	0.667	0.500	0.833
	LDA (1)	0.813	0.750	0.771	0.813	0.750
	PCA (2)	0.729	0.542	0.500	0.500	0.667
	PCA (70)	0.812	0.562	0.688	0.500	0.771
i-vectors (VoxCeleb)	None	0.542	0.563	0.521	0.625	0.625
	LDA (1)	0.542	0.521	0.521	0.542	0.521
	PCA (2)	0.750	0.625	0.563	0.708	0.729
	PCA (10)	0.562	0.542	0.438	0.583	0.562
i-vectors (Pitt)	None	0.417	0.521	0.521	0.438	0.542
	LDA (1)	0.417	0.542	0.542	0.417	0.542
	PCA (2)	0.667	0.583	0.708	0.604	0.646
	PCA (20)	0.583	0.542	0.583	0.521	0.479
i-vectors (VoxCeleb + Pitt)	None	0.458	0.521	0.500	0.500	0.563
	LDA (1)	0.458	0.542	0.542	0.458	0.542
	PCA (20)	0.458	0.563	0.604	0.458	0.479
x-vectors (VoxCeleb)	None	0.604	0.500	0.500	0.563	0.521
	LDA (1)	0.604	0.604	0.604	0.604	0.604
	PCA (2)	0.625	0.563	0.563	0.625	0.542
	PCA (40)	0.479	0.417	0.562	0.458	0.479
x-vectors (Pitt)	None	0.500	0.479	0.417	0.563	0.583
	LDA (1)	0.500	0.542	0.542	0.500	0.542
	PCA (40)	0.521	0.563	0.521	0.458	0.542
x-vectors (VoxCeleb + Pitt)	None	0.563	0.604	0.479	0.521	0.583
	LDA (1)	0.563	0.521	0.521	0.563	0.521
	PCA (40)	0.500	0.458	0.646	0.479	0.563

The highest-performing text classifiers outperformed the baseline text classifier, which was an LDA classifier trained on CLAN features with an average accuracy of 0.75. The highest-performing audio classifiers did not outperform the baseline audio classifier, which was an LDA classifier trained on ComParE openSMILE features with an average accuracy of 0.625.

### 3.2. MMSE Prediction

#### 3.2.1. Cross-Validation

For the MMSE prediction task, one of the speakers in the training set did not have an MMSE score and was excluded from training. Each of the regressors was evaluated on the challenge training set using LOSO cross-validation, where there was no speaker overlap between the training and test sets for each split. Each model was trained and tested at the utterance level, where an MMSE score was predicted for each utterance. Then the predicted MMSE scores of the utterances belonging to a patient were averaged to assign one MMSE score to that patient. Lastly, the RMSE between the predicted and ground truth MMSE scores was computed.

The average RMSE scores for each feature type can be seen in Table 5. For all of the features, the LR regressor trained on LDA-reduced features performed the same as the LR regressor trained on features with no dimensionality reduction. Although there was no difference in performance, results are included for completeness.

**Table 5:** LOSO RMSE scores for each of the classifiers. The results for the best-performing models for each feature type are red.

**Features**	**Dim. Red. (n_comp)**	**LR**	**DT**	**1NN**	**SVM**	**GradBoost**
LIWC	None	10.067	5.766	5.626	6.083	4.014
	LDA (23)	8.928	8.738	5.224	6.195	7.654
	PCA (20)	4.436	5.383	5.364	6.057	4.640
BERT	None	5.111	5.984	4.953	6.111	5.407
	LDA (23)	5.111	6.571	5.805	6.275	6.701
	PCA (2)	6.304	5.628	5.851	6.187	6.034
BERT + LIWC	None	9.475	4.956	4.752	5.919	4.050
	LDA (23)	8.515	8.038	5.285	6.821	7.234
	PCA (20)	4.574	5.228	5.680	5.165	4.509
BERT + CLAN	None	4.810	6.265	4.728	6.009	4.100
	LDA (23)	4.810	5.700	4.988	6.173	5.447
	PCA (20)	3.991	5.459	4.842	5.254	3.969
BERT + LIWC + CLAN	None	13.877	5.533	4.420	5.846	4.190
	LDA (23)	5.243	5.398	5.482	6.477	5.031
	PCA (20)	3.774	5.701	5.023	4.966	4.201
word vectors	None	5.294	5.467	5.204	6.146	5.684
	LDA (23)	5.294	5.158	4.967	5.936	5.228
	PCA (2)	6.359	6.061	5.958	6.148	6.241
	PCA (70)	5.419	5.561	4.981	6.177	5.516
i-vectors (VoxCeleb)	None	6.323	6.477	6.612	6.444	6.461
	LDA (23)	6.323	6.366	6.384	6.279	6.443
	PCA (2)	6.576	6.431	6.361	6.290	6.421
	PCA (10)	6.412	6.507	6.524	6.265	6.264
i-vectors (Pitt)	None	6.545	6.850	6.239	6.281	6.513
	LDA (23)	6.545	6.524	6.307	6.244	6.499
	PCA (2)	6.624	6.606	6.484	6.323	6.598
	PCA (20)	6.523	6.575	6.577	6.207	6.511
i-vectors (VoxCeleb + Pitt)	None	6.298	6.363	6.545	6.243	6.445
	LDA (23)	6.298	6.399	6.110	6.231	6.459
	PCA (20)	6.502	6.558	6.655	6.256	6.475
x-vectors (VoxCeleb)	None	6.424	6.400	6.208	6.400	6.369
	LDA (23)	6.424	6.478	6.493	6.162	6.413
	PCA (2)	6.618	6.767	6.531	6.381	6.634
	PCA (40)	6.246	6.320	6.517	6.329	6.378
x-vectors (Pitt)	None	6.310	6.534	6.445	6.405	6.504
	LDA (23)	6.310	6.073	6.403	6.245	6.318
	PCA (40)	6.471	6.456	6.181	6.369	6.474
x-vectors (VoxCeleb + Pitt)	None	6.385	6.268	6.394	6.401	6.386
	LDA (23)	6.385	6.379	6.230	6.170	6.442
	PCA (40)	6.296	6.433	6.411	6.288	6.467

The best-performing regressor trained on text features was the LR regressor trained on BERT embeddings combined with LIWC and CLAN features with PCA dimensionality reduction applied, which had an RMSE score of 3.774. The best-performing regressor trained on audio features was the DT regressor trained on x-vectors that were extracted using a system pre-trained on Pitt. LDA dimensionality reduction was applied and the RMSE score was 6.073.

The best-performing text regressors for every feature type, except for BERT embeddings and word vectors, performed better than the baseline text regressor that was evaluated using LOSO on the training set, which had an RMSE score of 4.38. The best-performing audio regressors for every feature type performed better than the baseline audio regressor that was evaluated using LOSO on the training set, which had an RMSE score of 7.28.

#### 3.2.2. Held-Out Test Set

The RMSE scores on the test set for each of the regressors can be seen in Table 6. The best-performing text regressor was the grad-boost regressor trained on BERT embeddings combined with CLAN features with PCA dimensionality reduction applied, which had an RMSE score of 4.560. The best-performing audio regressor was the 1NN regressor trained on i-vectors extracted using a system pre-trained on VoxCeleb and Pitt with LDA dimensionality reduction applied, which had an RMSE score of 5.694.

**Table 6:** RMSE scores for classifiers evaluated on the test set. The results for the best-performing models during cross-validation are red.

**Features**	**Dim. Red. (n_comp)**	**LR**	**DT**	**1NN**	**SVM**	**GradBoost**
LIWC	None	36.974	7.303	6.403	6.465	4.862
	LDA (23)	12.286	9.657	7.388	6.313	8.365
	PCA (20)	4.422	5.967	5.990	6.431	4.383
BERT	None	5.365	5.640	4.923	6.169	4.883
	LDA (23)	5.365	7.515	6.017	6.253	7.373
	PCA (2)	5.661	5.858	6.287	6.067	5.691
BERT + LIWC	None	34.420	7.127	5.021	6.103	5.037
	LDA (23)	14.905	8.624	5.742	7.189	6.561
	PCA (20)	4.872	7.078	5.159	4.895	4.404
BERT + CLAN	None	4.991	7.218	4.515	6.097	4.901
	LDA (23)	4.991	6.523	5.600	6.422	6.660
	PCA (20)	4.764	7.577	6.413	5.218	4.560
BERT + LIWC + CLAN	None	15.465	6.112	4.811	6.023	4.724
	LDA (23)	8.110	6.500	5.753	6.887	6.021
	PCA (20)	4.800	6.196	5.532	4.794	5.087
word vectors	None	4.714	5.280	5.129	6.147	5.361
	LDA (23)	4.714	5.111	5.344	6.063	4.955
	PCA (2)	5.732	6.452	5.992	6.129	5.803
	PCA (70)	4.785	5.700	5.237	6.169	5.271
i-vectors (VoxCeleb)	None	6.600	6.305	6.269	6.161	6.396
	LDA (23)	6.600	7.056	6.360	6.461	6.820
	PCA (2)	6.194	6.514	6.546	5.999	6.237
	PCA (10)	6.335	6.840	6.298	6.110	6.386
i-vectors (Pitt)	None	6.530	6.622	6.758	6.142	6.170
	LDA (23)	6.530	6.712	6.133	5.956	6.473
	PCA (2)	6.225	6.827	6.370	6.151	6.342
	PCA (20)	6.257	6.278	6.110	6.199	6.252
i-vectors (VoxCeleb + Pitt)	None	6.292	6.042	7.391	6.158	6.145
	LDA (23)	6.292	6.567	5.694	5.905	6.407
	PCA (20)	6.316	6.439	6.607	6.168	6.431
x-vectors (VoxCeleb)	None	6.559	6.665	6.401	6.094	6.309
	LDA (23)	6.559	6.289	6.261	6.085	6.312
	PCA (2)	6.167	6.669	6.566	6.089	6.164
	PCA (40)	6.358	6.058	6.189	6.115	6.160
x-vectors (Pitt)	None	6.428	6.483	6.563	6.287	6.333
	LDA (23)	6.428	6.462	6.314	6.097	6.423
	PCA (40)	6.424	6.506	6.499	6.322	6.370
x-vectors (VoxCeleb + Pitt)	None	6.644	6.622	6.338	6.096	6.208
	LDA (23)	6.644	6.450	6.188	6.059	6.466
	PCA (40)	6.173	6.640	6.488	6.123	6.204

The highest-performing text regressor outperformed the baseline text regressor, which was a DT regressor trained on CLAN features with an RMSE score of 5.20. The highest-performing audio regressor outperformed the baseline audio regressor, which was a DT regressor trained on Multi-resolution Cochleagram (MRCG) openSMILE features that had an RMSE score of 6.14.

### 3.3. Effects of Education and the Severity of Cognitive Impairment

In order to explore what effect the severity of cognitive impairment and education level had on the classification and MMSE prediction results, the best-performing text and audio models from both tasks were evaluated on smaller subsets of the test set that were split based on education level and MMSE score. According to the Alzheimer's Association (2020), an MMSE score of 20–24 corresponds to mild dementia, 13–20 corresponds to moderate dementia, and a score <12 is severe dementia. This information was used to create 4 groups of cognitive severity: healthy (MMSE score ≥25), mild dementia (MMSE score of 20–24), moderate dementia (MMSE score of 13–19), and severe dementia (MMSE score ≤12). The ranges set by the Alzheimer's Association were slightly modified to have unique boundary values.

For education level, the majority of patients had 12 years of education (likely equivalent to completing high school). Because the test set is small, we wanted to limit our experiments to a small number of groups. For the reasons previously mentioned, one education group was for patients that had 12 years of education, another group was for patients with <12 years of education, and the last group included patients that had more than 12 years of education.

The text and audio models were trained on the full training set and then evaluated on each MMSE and education group separately by only testing on patients in the test set that belonged to a particular group. The classification and MMSE prediction results can be seen in Table 7. For the MMSE groups, the results showed that the best classification accuracy achieved using a text model was 1.000 and that accuracy was achieved when the SVM classifier was evaluated on patients with severe dementia. The best RMSE achieved using a text model was 3.234 and that RMSE was achieved when the GradBoost regressor was evaluated on healthy patients. For the audio models, the best classification accuracy was 0.750 and was achieved when the 1NN classifier was evaluated on patients with severe dementia. The best RMSE was 1.801 and was achieved when the 1NN was evaluated on patients with mild dementia.

**Table 7:** Test set accuracies and RMSE scores for different levels of cognitive deficiency and education.

		**Text**			**Audio**	
		**Classification**		**MMSE prediction**	**Classification**	**MMSE prediction**
	**Group (num. patients)**	**SVM**	**RF**	**GradBoost**	**1NN**	**1NN**
MMSE	Healthy (28)	0.857	0.714	3.234	0.500	4.679
	Mild Dementia (8)	0.750	0.750	3.777	0.625	1.801
	Moderate Dementia (8)	0.875	0.625	4.563	0.500	6.224
	Severe Dementia (4)	1.000	0.500	10.241	0.750	12.323
Education	<12 years (5)	0.800	0.600	7.448	1.000	9.329
	12 years (24)	0.792	0.833	4.128	0.458	5.080
	>12 years (19)	0.947	0.684	3.885	0.474	5.138

For the education groups, the best classification accuracy achieved using a text model was 0.947, when the SVM classifier was evaluated on patients with more than 12 years of education. The best RMSE was 3.885 and was achieved when the GradBoost model was evaluated on patients with >12 years of education. For the audio models, the best classification accuracy is 1.000 and was achieved when the 1NN was evaluated on patients with <12 years of education. The best RMSE was 5.080 and was achieved when the 1NN was evaluated on patients with 12 years of education.

## 4. Discussion

The held-out test set results for both tasks show that text classifiers trained on BERT embeddings and text regressors trained on BERT embeddings combined with CLAN features perform better than text classifiers/regressors trained on only CLAN features (baseline text feature set). The results also show that audio classifiers trained on x-vectors and i-vectors, extracted using systems that were pre-trained on VoxCeleb and Pitt data, do not perform better than audio classifiers trained on ComParE openSMILE features (baseline audio feature set). However, audio regressors trained on x-vectors and i-vectors do perform better than audio regressors trained on MRCG openSMILE features when (1) the x-vectors are trained on only out-of-domain data or a combination of in-domain data and out-of-domain data and (2) when i-vectors are trained on a combination of in-domain and out-of-domain data.

We also note that we achieved better test set results on the classification task and equal test set results on the MMSE prediction task using a pre-trained BERT model as a feature extractor as opposed to using BERT as a classifier and regressor as Balagopalan et al. (2020) did. We received classification test set results equal to the BERT results of Yuan et al. (2020), who also used a BERT model as a classifier and added encoded pauses to their training regime. Our results show that BERT embeddings can be used to achieve the BERT model performance of Yuan et al. (2020) without using the BERT model itself as a classifier and without using pause information. However, the results of Yuan et al. (2020) suggest that we could achieve even greater performance if we include pause information in our feature set.

### 4.1. I-Vector and X-Vector Systems

One possible explanation for the poor performance of the i-vectors and x-vectors on the classification task is the domain-mismatch between the VoxCeleb datasets and the ADReSS dataset. While the pre-trained model may have learned some general representations of speech from the VoxCeleb datasets, it is possible that the type of representations that the model learned were not helpful for distinguishing between the speech of AD and non-AD patients. The VoxCeleb dataset consists of speech extracted from YouTube videos of celebrities being interviewed. While there is variety in the age, race, and accent of the speakers in the VoxCeleb dataset, which may help improve the ability of a model to distinguish between speakers that differ in these qualities, the nature of the recordings (i.e., background noise, overlapping speech, etc.) varies significantly from the recording environment of the ADReSS data. There is also less variety in the types of speakers present in the ADReSS dataset: they are all within a certain age range and do not seem to have significantly different accents. Therefore, the benefits of the VoxCeleb datasets are not likely to help with the AD classification task and the difference in recording environments likely intensifies the domain-mismatch problem, leading to lower performance. It is possible that i-vectors and x-vectors pre-trained on a different dataset with less of a domain-mismatch would perform better.

The i-vectors extracted from a system that was only trained on Pitt data did not improve performance on the classification task compared to the i-vectors extracted from a system that was trained on VoxCeleb but did improve performance on the MMSE prediction task. Conversely, the x-vectors extracted from a system that was only trained on Pitt did improve performance on the classification task but did not improve performance on the MMSE prediction task. The i-vector and x-vector extractors that we pre-trained on a combination of VoxCeleb and Pitt data led to an improvement in performance on the MMSE prediction task, compared to the performance for i-vectors and x-vectors extracted from a system trained on VoxCeleb. The x-vector performance also improved on the classification task. This shows that a small amount of in-domain data can improve i-vector and x-vector performance for the MMSE prediction task. When choosing between training i-vector and x-vector extractors on a large amount of out-of-domain data, a small amount of in-domain data, or a combination of both, the results suggest that it is best to train on a combination of both.

### 4.2. Pros and Cons of Linguistic Features

The highest-performing models for both tasks were trained on linguistic features (BERT embeddings). One benefit of using linguistic features is that punctuation can be included. This allows the model to use semantic and syntactical information, such as how often speakers are asking questions (“?” present in the transcript). Also, because the BERT model was pre-trained on BooksCorpus and English Wikipedia, the data that the pre-trained model saw was likely much more general than the VoxCeleb data and using text data meant that the model did not face the issue of the recording-environment mismatch.

However, there are some disadvantages associated with linguistic features. As discussed in the review paper of de la Fuente Garcia et al. (2020), transcript-free approaches to AD detection are better for generalizability and for protecting the privacy of the participants. In order to use linguistic features, the speech must be transcribed, meaning that linguistic features are worse for model generalizability and patient privacy. Using linguistic features depends on the use of automatic speech recognition (ASR) methods, which often have a low level of accuracy, or transcription methods, which can be costly and time-consuming.

Some linguistic features are also content- and language-dependent. There are linguistic features that are not content-dependent, such as word frequency measures, but it is difficult to automate the extraction of content-independent linguistic features (de la Fuente Garcia et al., 2020). For these reasons, it is important that future research explore using AD classification techniques that only require acoustic features.

### 4.3. Dimensionality Reduction

For the classification task, none of the highest-performing models had LDA dimensionality applied to the feature sets before training. As previously mentioned, the features were reduced to one dimension when LDA was applied. The results suggest that this dimensionality reduction was too extreme for the classification task and did not allow for enough information to be retained in the feature set. Conversely, the majority of the highest-performing classifiers had PCA dimensionality reduction applied to the feature sets before training. This suggests that applying PCA dimensionality reduction to the features before training can be useful for AD classification.

For the MMSE prediction task, the features were reduced to 23 dimensions when LDA was applied. Because the dimension was larger, LDA was more useful for this task. The best-performing audio model had LDA dimensionality reduction applied. PCA dimensionality reduction was also applied for some of the best-performing models, including the top-performing text model. This suggests that applying LDA and PCA dimensionality reduction to the features before training can be useful for MMSE prediction.

### 4.4. Group Evaluation

The evaluation results for different MMSE and education groups showed that certain MMSE groups can be classified more accurately (healthy, moderate dementia, and severe dementia) while others (mild dementia) are more difficult to classify. This seems very reasonable, as it is expected that more severe forms of dementia would be more easily distinguishable from healthy patients. Also, MMSE scores are predicted least accurately when evaluated on patients with severe dementia, regardless of the type of features used (text or audio).

The education results for the best-performing text-based model showed that patients with more than 12 years of education can be classified with high accuracy (0.947), while patients with exactly 12 years (0.792) and <12 years (0.800) of education are more difficult to classify and are classified with similar accuracy. The MMSE scores of patients with >12 years of education were predicted with the most accuracy.

These results provide some insight into which types of features are best for different levels of dementia and education for the classification and MMSE prediction tasks. However, it is important to note that the evaluation set is small, with as little as four speakers in certain groups (severe dementia). Therefore, these findings may not translate well to larger datasets.

### 4.5. Conclusions

In this paper, audio and text-based representations of speech were extracted from the ADReSS dataset for the AD classification and MMSE prediction tasks. Different dimensionality reduction techniques were applied to the data before training and testing the classification and regression models to explore whether applying dimensionality reduction techniques improved performance on those tasks. LOSO cross-validation was used to evaluate each of the classifiers and regressors and the models were also evaluated on a held-out test set.

The best-performing text models in this paper outperform the baseline text models on both tasks and the best-performing audio models outperform the baseline on the MMSE prediction task. The audio results suggest that, given access to a large amount of out-of-domain data and a small amount of in-domain data, it is best to use a combination of both to train i-vector and x-vector extractors. The comparison of the dimensionality reduction techniques shows that applying PCA dimensionality reduction to the features before training a classifier can be helpful for this particular AD classification task and possibly for other similar health-related classification tasks. Also, applying LDA and PCA dimensionality reduction to the features before training a regressor can be helpful for MMSE prediction tasks. Lastly, the evaluation results on different MMSE and education groups show that patients with more severe forms of dementia (moderate and severe) and healthy patients are easier to classify than patients with mild dementia, whereas the MMSE scores of severe dementia patients are the most difficult to predict. Patients with more than 12 years of education are the easiest to classify and the MMSE scores of patients with >12 years of education are the easiest to predict.

For future work, it would be interesting to repeat the experiments, particularly the evaluation of audio and text models on MMSE and education groups, on a larger dataset to see whether the findings translate. Another interesting future direction would be relating our findings to apathetic symptoms. Previous research has shown that patients with moderate or severe forms of AD tend to be apathetic (Lueken et al., 2007). Signs of apathy include slow speech, long pauses, and changes in facial expressions (Seidl et al., 2012). These characteristics can be measured using standardized ratings and we can explore whether our findings are consistent with the findings related to other forms of cognitive decline that affect speech.

## Data Availability Statement

The data analyzed in this study is subject to the following licenses/restrictions: in order to gain access to the datasets used in the paper, researchers must become a member of DementiaBank. Requests to access these datasets should be directed to https://dementia.talkbank.org/.

## Author Contributions

R'mH contributed to the design and implementation of the research, to the analysis of the results, and to the writing of the manuscript. JG contributed to the design of the research and supervised the findings of this work. All authors contributed to the article and approved the submitted version.

## Conflict of Interest

The authors declare that the research was conducted in the absence of any commercial or financial relationships that could be construed as a potential conflict of interest.
